# Early Pregnancy Vitamin D Binding Protein Is Independently Associated with the Development of Gestational Diabetes: A Retrospective Cohort Study

**DOI:** 10.3390/jcm9072186

**Published:** 2020-07-10

**Authors:** Melinda Fernando, Stacey J. Ellery, Deborah de Guingand, Clara Marquina, Siew Lim, Cheryce L. Harrison, Helena J. Teede, Negar Naderpoor, Aya Mousa

**Affiliations:** 1 Monash Centre for Health Research and Implementation (MCHRI) and Centre of Cardiovascular Research and Education in Therapeutics (CCRET), School of Public Health and Preventive Medicine, Monash University, 43-51 Kanooka, Grove, VIC 3168, Australia; melinda.fernando@monash.edu (M.F.); clara.marquina@monash.edu (C.M.); siew.lim1@monash.edu (S.L.); cheryce.harrison@monash.edu (C.L.H.); helena.teede@monash.edu (H.J.T.); negar.naderpoor@monash.edu (N.N.); 2 The Ritchie Centre, Hudson Institute of Medical Research and Department of Obstetrics and Gynaecology, Monash University, 43-51 Kanooka, Grove, VIC 3168, Australia; stacey.ellery@hudson.org.au (S.J.E.); deborah.deguingand@hudson.org.au (D.d.G.)

**Keywords:** vitamin D binding protein, free vitamin D, bioavailable vitamin D, pregnancy, gestational diabetes mellitus, glucose tolerance

## Abstract

Background: Vitamin D-binding protein (VDBP) has been implicated in several adverse pregnancy outcomes either directly or indirectly via influencing the concentrations of biologically active vitamin D metabolites. However, human studies exploring these metabolites in pregnancy remain sparse. Here, we examine whether VDBP and total, free, and bioavailable 25-hydroxyvitamin D (25(OH)D) metabolites in early pregnancy are associated with subsequent adverse pregnancy outcomes. Methods: We conducted a retrospective analysis of 304 pregnant women in early pregnancy (<20 weeks gestation). The demographic characteristics, anthropometric data, and total 25(OH)D were measured and plasma or serum samples were collected and bio-banked. Using these samples, we measured VDBP (polyclonal ELISA) and albumin (automated colorimetry), and calculated free and bioavailable 25(OH)D using validated formulae. Pregnancy outcomes were derived from scanned medical records. Regression models were used to analyse the relationships between vitamin D metabolites in early pregnancy and subsequent pregnancy outcomes (gestational diabetes mellitus (GDM), pre-eclampsia, preterm birth), with adjustment for predetermined clinically relevant maternal factors including age, body mass index (BMI), and ethnicity. Results: Lower VDBP concentrations were associated with higher glucose levels and a greater likelihood of developing GDM at 26–28 weeks gestation (odds ratio [OR] (95% CI) = 0.98 (0.97,0.99), *p* = 0.015). This finding remained significant after adjustment for maternal covariates including age, BMI, and ethnicity (*β* = −0.003, *p* = 0.03). Lower total, free and bioavailable 25(OH)D, but not VDBP, were associated with a shorter length of gestation, but only the relationship with total 25(OH)D remained significant after adjustment for the above maternal covariates (*β* = 0.02, *p* = 0.006). Conclusions: This is the first study to examine VDBP, and total, free and bioavailable 25(OH)D in relation to pregnancy outcomes in a well characterised multi-ethnic cohort of pregnant women. Our findings show that VDBP and total 25(OH)D are associated with GDM and length of gestation, respectively; however, further investigations using large-scale prospective studies are needed to confirm our findings.

## 1. Introduction

Vitamin D has a well established role in calcium homeostasis and bone mineralisation, and has been shown to contribute to other biological processes including inflammation [1], immunoregulation and cardiometabolic health [2,3]. During pregnancy, there is an increased requirement for vitamin D to meet heightened physiological and metabolic demands in the mother and to support fetal skeletal bone mineralisation [4]. Vitamin D deficiency in pregnancy has been linked with several adverse pregnancy outcomes including pre-eclampsia, gestational diabetes mellitus (GDM) and preterm birth [2]. In particular, GDM is a common pregnancy complication closely linked to maternal obesity and its incidence has been linked to several lifestyle factors including nutritional inadequacies such as vitamin D deficiency [5]. However, published results are inconsistent and current classifications for vitamin D deficiency or optimal concentrations during pregnancy remain contentious [2,3].

Recent evidence suggests that vitamin D binding protein (VDBP) is critical for maintaining vitamin D homeostasis in pregnancy [6]. VDBP is the main carrier of vitamin D and its concentrations increase during pregnancy (mainly in response to rising oestrogen), altering the concentrations of, and the relationships between, total and free 25-hydroxyvitamin D (25(OH)D) [7]. Currently, total 25(OH)D concentrations are used to determine vitamin D status and to define deficiency [2]. Free or unbound 25(OH)D makes up less than 1% of total serum 25(OH)D, while bioavailable 25(OH)D, which includes a combination of free 25(OH)D and albumin-bound 25(OH)D (with much lower binding affinity) makes up approximately 10–15% of total 25(OH)D [6]. The ‘free hormone hypothesis’ posits that only free steroid hormones are physiologically active since their lipophilic ability enables their passive diffusion across cell membranes [6]. This demonstrates the potential importance of VDBP since it directly regulates the circulating amounts of the biologically active free vitamin D [6], as well as preventing the urinary loss of vitamin D [8]. However, existing studies exploring vitamin D in pregnancy have focused primarily on total 25(OH)D as a single measure of vitamin D status, which may be inadequate for representing functional vitamin D status.

Independent of its role in vitamin D transport and regulation, VDBP has been shown to directly influence several biological processes that are often exacerbated in the pregnant state, including immunoregulation, inflammation, glucose metabolism, and the regulation of blood pressure [9]. Studies report that VDBP is enhanced during pro-inflammatory states and that it maintains an environment of tolerance to paternal and foetal tissue and their accompanying alloantigens [6]. Despite the increased recognition of the possible multifunctional role of VDBP in promoting a healthy pregnancy, human studies examining the relationships between VDBP and pregnancy outcomes remain sparse. These studies have been reviewed by our group [10] and others [6], highlighting key limitations in the evidence including small sample sizes, minimal ethnic diversity, and the lack of adjustment for potential confounding variables (obesity, age, ethnicity, lifestyle factors), all of which can affect both VDBP levels and pregnancy outcomes [11,12,13]. Ethnicity in particular is associated with genetic polymorphisms which influence VDBP concentrations, binding capacity, and function [14,15,16]. Previous studies are also limited by the use of monoclonal immunoassays to measure VDBP, which have a different selective affinity for VDBP genotypes and are considered less accurate than polyclonal assays for measuring VDBP and subsequently calculating free 25(OH)D [17]. Studies addressing these knowledge gaps may be useful to enhance our understanding of the complex vitamin D network and to potentially identify biomarkers to facilitate risk prediction and prevention of adverse pregnancy outcomes.

Therefore, we aimed to examine whether maternal concentrations of VDBP, and total, free and bioavailable 25(OH)D metabolites in early pregnancy were associated with subsequent pregnancy outcomes in a well characterised multi-ethnic cohort of pregnant women. We tested the hypothesis that VDBP concentrations in early pregnancy will be correlated with adverse pregnancy outcomes either directly and/or indirectly via influencing free and bioavailable 25(OH)D concentrations.

## 2. Experimental Section

### 2.1. Study Design and Population

This study is a longitudinal retrospective cohort study of 304 pregnant women. Datasets and pre-collected bio-banked samples from two separate populations were combined and analysed in this study and their respective inclusion criteria are described in Table 1. The first population was derived from the healthy lifestyle in pregnancy (HLP) study cohort [18], which comprised 228 women initially recruited from Monash Health (three large tertiary hospitals in Melbourne, Australia). These women were recruited during early pregnancy (<15 weeks gestation) for a randomised controlled trial (RCT) which aimed to prevent excess gestational weight gain in women classified as high risk for the development of GDM, based on a validated risk prediction tool [19]. Of these women, 103 were in the HLP control group (receiving standard care) and the data and samples for these 103 control group women were sought for the present study.

The second population, the creatine and pregnancy outcomes (CPO) cohort, were a low risk pregnancy group (participants with significant medical or obstetric history were excluded; Table 1). This cohort was recruited in early pregnancy (<20 weeks gestation), also from Monash Health, for a prospective observational study aimed at characterising creatine homeostasis in low-risk pregnancies [20]. The CPO low-risk pregnancy cohort recruited 282 pregnant women, of which 18 subsequently withdrew or were excluded, and 264 remained involved until the study conclusion.

Of the 103 women in the HLP control cohort and the 264 in the CPO cohort, our current analysis utilised data and bio-banked samples from 91 and 213 women, respectively (total *n* = 304). The remaining women could not be included due to missing data, insufficient samples (for the laboratory analysis of VDBP and albumin) and/or unreliable assay measures.

### 2.2. Ethics

All participants provided informed written consent prior to participation and all the data were de-identified for use in this study. The HLP high-risk pregnancy cohort study was approved by the Monash Health Research Advisory and Ethics Committee (07216C) initially in 2008, and in 2019 for the current retrospective study (19674). For the low-risk CPO pregnancy cohort, ethics approval was obtained in 2015 from Monash Health (14140B) and Monash University (7785), and in 2019 for the current retrospective study (HREC/51952/MonH-2019-169657(v2)).

### 2.3. Sample and Data Collection

The data were collected at several stages in the two cohorts. The timepoints, data collected, and methods are summarised in Table 2. The maternal outcomes were determined by routine clinical assessments and derived for the purpose of this study from the Birthing Outcomes System (BOS) database which uses a standardized method of reporting perinatal data in Victoria, Australia [19]. Oral glucose tolerance test (OGTT) values at 26–28 weeks were used to diagnose GDM based on the 2014 Australasian Diabetes in Pregnancy Society criteria [21]. Pre-eclampsia and preterm birth (<37 weeks gestation) were diagnosed based on the Royal Australia New Zealand College of Obstetricians and Gynaecologists guidelines [22].

### 2.4. Biochemical Analyses

#### 2.4.1. Archived Data (Serum Glucose, Total 25(OH)D)

As shown in Table 2 above, fasting glucose was measured in fasting serum samples collected in early pregnancy (<20 weeks), while the OGTT glucose levels (fasting, 1 h and 2 h) were measured in serum samples collected at 26–28 weeks gestation. These glucose measures were collected in accordance with routine care at Monash Health and were analysed by Melbourne Pathology and Monash Health Pathology using commercial enzyme-linked immunosorbent assays (ELISA) with the relevant quality control standards.

Total 25(OH)D concentrations were measured in early pregnancy serum samples (<20 weeks) by Monash Health Pathology using direct competitive chemiluminescent immunoassays on a LIAISON analyser (DiaSorin Inc., Stillwater, MN, USA), with inter- and intra-assay coefficients of variation (CVs) of <10% and <4%, respectively.

#### 2.4.2. VDBP Analysis

For the purpose of this study, we measured VDBP in samples which were stored at −80 °C in departmental bio-banks. The VDBP analyses were conducted using polyclonal competitive ELISA assays (Abcam ab108853), according to the manufacturer’s instructions. The samples were run in duplicate at random across nine plates, with a calibrator sample used to determine the inter-assay variability and normalise the measures across the plates. Before correction, the intra- and inter-assay CVs were <12% and <22%, respectively. We proceeded to calculate adjustment factors from the calibrator sample and conducted the analysis using the adjusted results, where appropriate.

#### 2.4.3. Albumin Analysis

Albumin was measured in order to calculate free and bioavailable 25(OH)D values using the formula by Bikle et al. [23]. The samples were analysed by Monash Health Pathology using an automated colorimetric method carried out on a Beckman Coulter AU5812 System.

### 2.5. Calculation of Free and Bioavailable 25(OH)D

Using the formula by Bikle et al. [23], free 25(OH)D was calculated based on the affinity binding constants for 25(OH)D with albumin and VDBP (6 × 10^5^ M^−1^ and 7 × 10^8^ M^−1^, respectively) as determined using centrifugal ultrafiltration dialysis. The calculations used for free and bioavailable vitamin D and the relevant unit conversions are summarised below:

$$VD_{free}=\frac{VD_{total}}{\left(1+\left(6\times 10^{5}\right)\times Alb\right)+\left(\left(7\times 10^{8}\right)\times VDBP\right)}$$

$$VD_{bio}=\left(1+\left(6\times 10^{5}\right)\times Alb\right)\times VD_{free}$$

where:

$VD_{free}\;$ = serum free 25(OH)D concentrations in mol/L;
$VD_{total}\;$ = serum total 25(OH)D concentration in mol/L;
$VD_{bio}\;$ = serum bioavailable 25(OH)D concentration in mol/L;
Alb  = serum albumin concentration in mol/L (albumin was measured in g/L and converted to mol/L using: g/L÷66430);
VDBP  = serum vitamin D binding protein concentration in mol/L (VDBP was measured in ug/mL and converted to mol/L using: $µg/\mathrm{mL}÷5.8\times 10^{7}$).

Free 25(OH)D is reported in pg/mL and was converted from mol/L to pg/mL using: mol/L × 0.4166 × 10^12^. Total and bioavailable 25(OH)D are reported in nmol/L and were converted from mol/L to nmol/L using: mol/L × 10^9^.

### 2.6. Statistical Analyses

The participant demographics, clinical and biochemical parameters including vitamin D metabolites are presented as mean ± standard deviation (SD) or frequencies (%). Normality was determined using Shapiro–Wilk tests and by visual inspection of histograms. Non-normally distributed variables including total, free, and bioavailable 25(OH)D metabolites were logarithmically transformed to the base 10 to meet the assumptions of parametric statistical tests prior to data analysis. Univariable associations between the continuous vitamin D metabolite concentrations and continuous or categorical demographic or biochemical parameters were analysed using Pearson’s correlations or chi-square tests. Univariable associations between vitamin D metabolites and continuous (e.g., length of gestation) or categorical pregnancy outcomes (e.g., binary ‘with GDM’ or ‘without GDM’) were analysed using general linear or simple logistic regression, respectively. Differences in the mean concentrations of the vitamin D metabolites between binary outcome groups (e.g., women with or without GDM) were explored using independent Student’s *t*-tests.

Multiple linear and logistic regression models were used to adjust for predetermined maternal characteristics considered to be clinically relevant to the outcomes, including maternal age, body mass index (BMI), and ethnicity. Exploratory analyses of additional covariates such as smoking status, parity, or previous history of GDM were included as appropriate. The statistical analyses were performed using Stata V.15.0 (StataCorp, College Station, TX, USA) and a two-tailed *p* < 0.05 was considered statistically significant.

## 3. Results

### 3.1. Sample Characteristics

Three hundred and four pregnant women were included in this study and the sample characteristics are presented in Table 3. There were no differences between the two cohorts of women (HLP and CPO) in any demographic or anthropometric characteristics at early pregnancy (<20 weeks gestation) including age, BMI, parity, and ethnicity (all *p* > 0.05). The mean age of the participants was 31.4 ± 4.2 years (mean ± SD) and 46% were primiparous. The sample was ethnically diverse, with approximately 43% being of non-Caucasian background (Table 3). Approximately 28.3% of participants were classified as overweight (BMI ≥ 25 kg/m^2^) and 25.6% as obese (BMI ≥ 30 kg/m^2^).

At early pregnancy, the mean level of serum total 25(OH)D was 54.8 ± 20.2 nmol/L (22.0 ± 8.1 ng/mL). Only 14.5% of the sample had sufficient levels of vitamin D (total 25(OH)D ≥75 nmol/L or ≥30 ng/mL) according to the US Endocrine Society guidelines [24], with 46.8% having insufficient levels (50–74.9 nmol/L or 20–29.9 ng/mL), 30.3% having deficient levels (25–49.9 nmol/L or 10–19.9 ng/mL), and 8.4% deemed severely deficient (<25 nmol/L or <10 ng/mL). There was a total of 55 (19.4%) cases of GDM, 10 cases (3.4%) of pre-eclampsia, and 16 cases (5.4%) of preterm birth (Table 3).

### 3.2. Univariable Analyses of Vitamin D Metabolites and Demographic and Anthropometric Variables

The correlations between VDBP and total, free, and bioavailable 25(OH)D metabolites with demographic and anthropometric parameters are presented in Table 4. Higher maternal age was correlated with lower VDBP and higher free and bioavailable 25(OH)D concentrations (Table 4). A higher BMI correlated with lower VDBP and total 25(OH)D but was not associated with free or bioavailable 25(OH)D (Table 4). There were no associations between vitamin D metabolites and any other demographic characteristics (all *p* > 0.05).

In examining correlations between the vitamin D metabolites, VDBP was inversely associated with free and bioavailable 25(OH)D (both *r* = −0.6, *p* < 0.001), but there was no association between VDBP and total 25(OH)D (*p* = 0.8). Total 25(OH)D concentration was positively associated with free and bioavailable 25(OH)D (both *r* = 0.7, *p* < 0.001) and there was a positive correlation between the free and bioavailable 25(OH)D metabolites (*r* = 0.9, *p* < 0.001).

### 3.3. Univariable and Multivariable Analyses of Vitamin D Metabolites and Biochemical Variables

**Figure 1:**
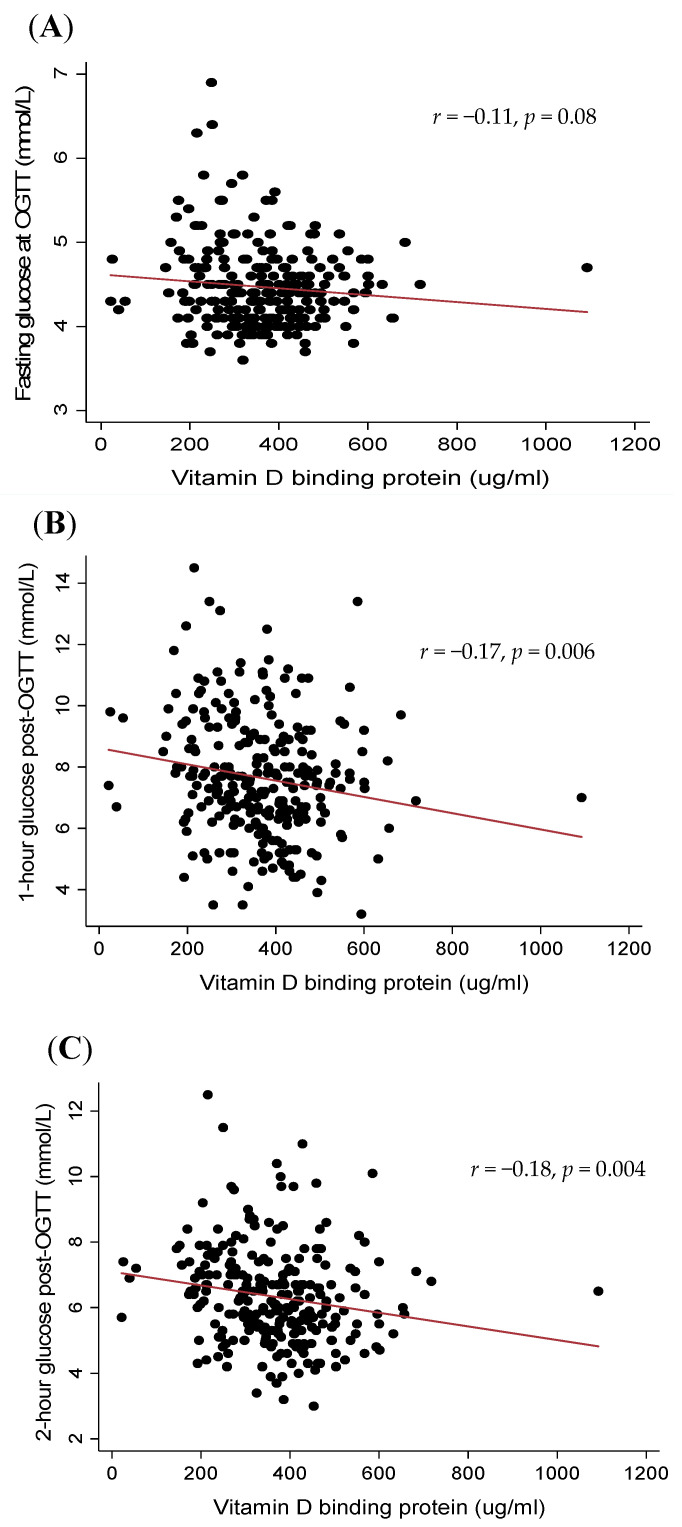
Scatterplots illustrating the correlations between vitamin D binding protein with fasting glucose (**A**); 1 h glucose (**B**); and 2 h glucose (**C**) post-oral glucose tolerance test (OGTT).

**Figure 2:**
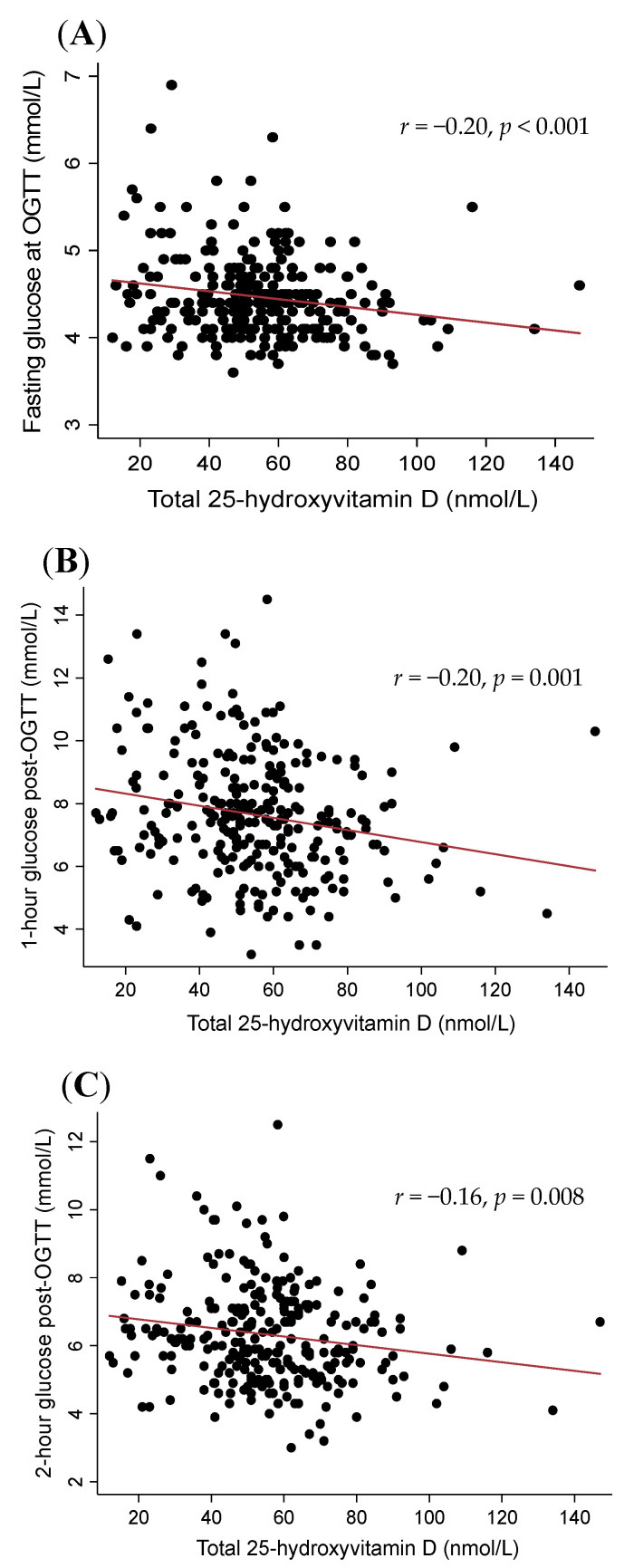
Scatterplots illustrating the correlations between total 25(OH)D with fasting glucose (**A**); 1 h glucose (**B**) and 2 h glucose (**C**) post-oral glucose tolerance test (OGTT).

Associations between vitamin D metabolites and glucose measures are presented in Table 4 and Figure 1 and Figure 2. Higher VDBP concentrations in early pregnancy were associated with lower 1 h and 2 h glucose post-OGTT at 26–28 weeks (both *p* < 0.01) but not with fasting glucose at OGTT (*p* = 0.08, Table 4 and Figure 1). Similarly, higher total 25(OH)D concentrations in early pregnancy were associated with lower fasting glucose, as well as lower 1 h and 2 h glucose during OGTT at 26–28 weeks gestation (Table 4 and Figure 2). Free and bioavailable metabolites were not associated with any glucose measures (Table 4).

After adjusting for maternal covariates including age, BMI and ethnicity, VDBP remained associated with 1 h (*β* = −1.7, *p* = 0.01) and 2 h glucose (*β* = 1.4, *p* = 0.004), but not with fasting glucose (*p* = 0.2). A higher total 25(OH)D remained associated with fasting glucose (*β* = −0.5, *p* = 0.008) but was no longer associated with 1 h or 2 h glucose post-OGTT, although the results were borderline non-significant (*p* = 0.06 and *p* = 0.07, respectively). There were no significant associations between the free or bioavailable 25(OH)D metabolites with the glucose measures in any of the multivariable models (all *p* > 0.1).

### 3.4. Univariable Analyses of Vitamin D Metabolites and Pregnancy Outcomes

The relationships between vitamin D metabolites and the pregnancy outcomes are presented in Table 5. VDBP was significantly associated with GDM (Table 5), whereby women who developed GDM had a significantly lower mean VDBP concentration in early pregnancy compared with the women who did not develop GDM (325.4 ± 109.1 µg/mL versus 371.3 ± 127.7 µg/mL respectively, *p* = 0.01). VDBP was not correlated with pregnancy-induced hypertension, pre-eclampsia, placental abnormalities, pre-labour rupture of membranes (PROM) or preterm birth (all *p* > 0.05; Table 5).

Total 25(OH)D concentrations were also associated with GDM (Table 5), whereby women with GDM had a lower total 25(OH)D concentration compared with the women without GDM (55.6 ± 19.2 versus 50.7 ± 24.4 nmol/L, respectively, *p* = 0.04). Higher total 25(OH)D concentrations in early pregnancy were also associated with a greater length of gestation (*β* = 0.02, *p* = 0.002), but not with any of the other outcomes measured (*p* = 0.06 for pregnancy-induced hypertension; remaining outcomes *p* ≥ 0.1; Table 5). Free and bioavailable 25(OH)D were not associated with any of the measured pregnancy outcomes (all *p* > 0.05; Table 5). In an exploratory sub-analysis of the two cohorts separately, there were no associations between VDBP or any vitamin D metabolites with GDM or any pregnancy outcomes (all *p* > 0.05) in the high-risk cohort (HLP, *n* = 91) or the low-risk cohort (CPO; *n* = 213).

### 3.5. Multivariable Analyses of Vitamin D Metabolites and Pregnancy Outcomes

Results of the multivariable logistic and linear regression analyses for the pregnancy outcomes are shown in Table 6. These models were adjusted for predetermined covariates based on clinical relevance to the pregnancy outcomes, including maternal age, BMI, and ethnicity. Additional variables including parity or previous history of GDM were adjusted for in further exploratory analyses.

VDBP remained significantly associated with GDM in the fully adjusted model with age, BMI and ethnicity as covariates (*β* = −0.003, *p* = 0.03), as well as after adjusting for parity, smoking status, or previous history of GDM in additional exploratory analyses (*β* = −0.003, *p* = 0.03 in all the models). There were no associations between VDBP and other outcomes including pregnancy-induced hypertension, pre-eclampsia, PROM, preterm birth or the length of gestation in any of the multivariable models (Table 6).

A lower total 25(OH)D concentration remained associated with a higher risk of GDM after adjusting for age (*p* = 0.01) and BMI (*p* = 0.04), but this relationship was attenuated after adjusting for ethnicity (*p* = 0.1, Table 6). A higher total 25(OH)D concentration remained associated with a greater length of gestation after adjustment for age (*p* = 0.001), BMI (*p*= 0.001) and ethnicity (*p* = 0.006; Table 6), but was not associated with any other outcomes (all *p* > 0.05).

Higher free and bioavailable 25(OH)D concentrations were associated with a greater length of gestation in the models adjusted for age and BMI (*p* = 0.03 for all); however, these were attenuated upon adjustment for ethnicity (Table 6). Neither free 25(OH)D nor bioavailable 25(OH)D were associated with the other outcomes measured in any of the multivariable models (all *p* > 0.05; Table 6).

## 4. Discussion

### 4.1. Summary of Results

To our knowledge, this is the first study to assess VDBP and total, free and bioavailable 25(OH)D concentrations in a well characterised multi-ethnic cohort of pregnant women. Moreover, although the association between total 25(OH)D and GDM has been extensively reported previously [25,26,27], this is the first study to report an association between VDBP and GDM. Our results show that women with lower concentrations of VDBP at early pregnancy had higher glucose levels at OGTT and a greater likelihood of developing GDM at 26–28 weeks gestation compared with women with higher VDBP concentrations, irrespective of free and bioavailable 25(OH)D concentrations. Lower total 25(OH)D concentrations in early pregnancy were also associated with higher glucose and a greater risk of GDM. These relationships remained significant after adjusting for age and BMI but only the relationship with VDBP persisted after the additional adjustment for ethnicity, demonstrating the robustness of the relationship between VDBP and GDM. We also show that higher total, free, and bioavailable 25(OH)D were associated with a greater length of gestation, but only the relationship with total 25(OH)D persisted in the fully-adjusted model.

### 4.2. Comparison with Previous Literature and Potential Mechanisms

In the present study, glucose concentrations post-OGTT and GDM were shown to be inversely associated with not just total 25(OH)D concentration, which has been widely reported [25,27,28,29] though inconsistently [30,31,32], but also with VDBP. This novel relationship between VDBP and GDM has not previously been reported with the single earlier study examining VDBP in women with and without GDM, finding no association [33], contrary to our findings. The lack of association reported by Xia et al. [33] may be attributed to their use of monoclonal assays to measure VDBP since, as acknowledged by the authors, these assays have been criticised for their lack of sensitivity to several genetically determined isoforms found in individuals who carry the *GC-1F* allele (predominantly in certain ethnicities) [17]. Conversely, our study utilised polyclonal assay technology, which is less affected by genetic and/or ethnic variations and this may explain the discrepant findings between the two studies. Based on our findings, it would be expected that low VDBP would correspond with higher free or bioavailable 25(OH)D concentrations, and that these metabolites would also be associated with a higher risk of GDM. This would be inconsistent with previous literature showing that free or bioavailable metabolites are associated with positive outcomes in several conditions [34,35], as well as the aforementioned studies and meta-analyses showing that higher total 25(OH)D is associated with a lower risk of GDM [25,27,28,29]. Indeed, higher total 25(OH)D in our study was associated with a reduced risk of GDM, consistent with previous literature, but while VDBP was correlated with free and bioavailable 25(OH)D, we found no relationships between these metabolites and GDM. The absence of these latter associations may be due to our use of calculated free and bioavailable 25(OH)D, rather that the direct measurement of these metabolites. It is also possible that changes in VDBP during pregnancy do not influence the bioavailability of vitamin D, although evidence on this has been inconsistent. One study [36] reported lower bioavailable 25(OH)D in pregnant women compared to non-pregnant women, while another study [37] found no differences in free 25(OH)D between pregnant and non-pregnant women. In a study of 40 women across four timepoints in pregnancy [38], free 25(OH)D was reduced at all timepoints but there were no differences in total 25(OH)D compared with non-pregnant controls. Importantly, none of these studies assessed whether maternal free or bioavailable metabolites were associated with adverse pregnancy outcomes. A compensatory mechanism has been posited, whereby VDBP may exhibit a lower affinity for its metabolites during pregnancy to enable the level of free or bioavailable 25(OH)D to remain balanced, despite an increase in VDBP [39]. However, this altered affinity cannot be confirmed with the present data and requires further exploration.

There are several potential mechanisms underlying the association between VDBP and GDM. One explanation is that VDBP is the primary carrier protein and influences the proportions of the biologically active free and bioavailable 25(OH)D metabolites. This would mean that both higher VDBP and proportionally lower free and bioavailable 25(OH)D concentrations would be associated with GDM risk. As mentioned above, this was not demonstrated in our study or in other earlier studies [33,40]. We speculate that the influence of VDBP on GDM may be direct and independent of its role as a vitamin D carrier protein. For instance, VDBP may influence GDM directly via driving the actin scavenger system during inflammation and cell injury [41]. Recent evidence suggests that VDBP-actin complexes may serve as more than benign by-products of cell injury, potentially having an important role in both the mediation and resolution of inflammation and tissue injury [42]. Given that GDM and insulin resistance are underscored by inflammation [43,44], higher VDBP concentrations may mediate chronic low-grade inflammation, thereby influencing glucose metabolism and potentially reducing the risk of GDM. Another mechanism pertains to the interplay between VDBP, oestrogen and insulin. Both VDBP and insulin resistance are independently influenced by the rising oestrogen levels in pregnancy [45,46]. Studies show that VDBP increases exponentially, corresponding with the rise and peaks of oestrogen in pregnancy [45], alongside a documented decline in insulin sensitivity which is mediated by several factors including the increase in oestrogen [46]. Animal-based studies report that oestrogen counteracts the impact of insulin deficiency on vitamin D metabolism, and vice versa, supporting the likelihood of a complex relationship between VDBP and insulin resistant and deficient states such as GDM [47]. Sex hormones including oestrogen were not measured in the present study and the interplay between these hormones with VDBP and GDM warrants further exploration. VDBP may also be directly related to insulin regulation, as shown in a study of women with and without polycystic ovary syndrome, where VDBP levels were negatively correlated with serum insulin in both groups independent of the influence of sex hormones including oestrogen and androgen levels [48]. On the other hand, genetic studies have linked *GC* allelic variants of VDBP with serum glucose levels and the development of diabetes [49,50]. Although we did not explore genetic variants in the present study, the relationship between VDBP and GDM persisted after adjustment for ethnicity, suggesting that it may extend beyond ethnic or genetic differences.

We report that none of the vitamin D metabolites or VDBP were associated with placental disorders including pre-eclampsia or pregnancy-induced hypertension. Of the limited literature exploring VDBP in pregnancy, placental disorders, particularly pre-eclampsia, is an area which has been more extensively investigated. The role of VDBP in the actin-scavenger system of inflammation and tissue injury, independent of its involvement in the vitamin D metabolic system, has been consistently implicated in placental dysfunction [51,52]. VDBP displays a high binding affinity for free actin released as a result of cell death or tissue injury (which underlies the pathology of pre-eclampsia and other placental disorders [53]), and actively sequesters these products of tissue injury [54]. The increased expression of VDBP in pro-inflammatory states such as pregnancy or pre-eclampsia may be a mechanism by which actin debris is cleared. However, current findings lack consistency, with one study [51] reporting that VDBP is upregulated in women with early-onset pre-eclampsia and another reporting the decreased expression of VDBP in states of oxidative stress associated with the pathophysiology of pre-eclampsia [55]. These studies had a small number of pre-eclampsia cases (five and 11, respectively), as did the present study with only 10 cases, and hence were likely underpowered to identify true associations. Larger-scale studies to clarify the relationship between VDBP and placental disorders including preeclampsia are warranted.

The vitamin D metabolites analysed in this study, including VDBP, were not associated with preterm birth or PROM. Preterm birth and PROM have previously been linked to increased levels of VDBP in cervicovaginal fluid (CVF) [56], with a study by Liong et al. [57] finding that the VDBP/albumin CVF ratio was a more efficacious and precise biomarker than fetal fibronectin (fFN), the current gold standard, for predicting impending preterm labour. Overall, much of the existing research has explored VDBP levels in CVF, which may explain the discrepancies with our findings since VDBP in this study was measured in plasma/serum. The lack of significant findings for both PROM and preterm birth may also be due to insufficient statistical power, given the study sample size and the relatively small proportion of cases, particularly for preterm birth (*n* = 16; 5%). Nevertheless, the length of gestation was significantly associated with total 25(OH)D as well as with free and bioavailable 25(OH)D, although the latter were attenuated after accounting for ethnicity in the multivariable models. The positive association between the length of gestation and total 25(OH)D has been reported previously [58,59,60,61], indicating that higher levels of total 25(OH)D may indeed be related to delivery at a later gestation. However, the relationships between free and bioavailable 25(OH)D with length of gestation await further study.

Mechanistically, vitamin D deficiency may promote proximal muscle weakness, thus having a possible role in reducing the length of gestation via the initiation of early labour [62]. Vitamin D also induces antimicrobial and anti-inflammatory properties, which may increase gestation by reducing the risk of infection and membrane rupture [63,64]. The relationships between vitamin D metabolites and the length of gestation are intriguing and with the plausibility of these mechanisms, warrant further exploration, particularly in larger cohorts with sufficient statistical power.

### 4.3. Strengths and Limitations

Some limitations should be acknowledged. The observational study design precludes assessments of causality and potential confounding cannot be ruled out. Because this was a secondary post-hoc analysis of two previous studies where recruitment and data collection were complete, no additional data or samples could be collected and there was no formal power calculation. As noted earlier, despite combining two cohorts, our sample size is modest and may have been underpowered to detect the associations between VDBP or total, free or bioavailable 25(OH)D metabolites with some of the pregnancy outcomes analysed. The single measurement of vitamin D metabolites during early pregnancy (<20 weeks gestation) meant that we could not determine or adjust for temporal changes in vitamin D metabolites due to seasonality, sun exposure, supplement use or the dietary intake of vitamin D over the course of the pregnancy. The exact gestational week of data collection for each participant was not available, and including this in the statistical models may have influenced our results since both VDBP and BMI change over the course of pregnancy. We expect that the impact of this would be minimal, however, since data for this study were collected in early pregnancy, while it is known that VDBP and BMI change more drastically at the later stages of pregnancy. Nevertheless, our results should be interpreted in light of these important limitations. Only the maternal outcomes were examined and other birth or neonatal outcomes including those relevant to GDM such as neonatal birthweight or large-for-gestational-age should be assessed in future studies. Genetic differences and the potential contribution of vitamin D gene axis polymorphisms were not assessed in this study. We used Diasorin assays to measure the concentrations of total serum 25(OH)D rather than the gold-standard liquid chromatography mass-spectrometry method (LC/MS). Finally, free 25(OH)D was calculated rather than measured directly. Although the formula used in this study [23] has been shown to correlate well with direct measures of free 25(OH)D in most populations, some discrepancies have been identified, particularly in populations of African descent [65], of which there were three in this cohort. Further research utilising the newly developed and potentially superior ELISA for free 25(OH)D would be beneficial to corroborate our findings.

Notwithstanding these limitations, our study is the first to report a relationship between maternal VDBP and the development of GDM. The sample comprised a well characterized cohort of women at high and low risk of GDM, which allowed the examination of a diverse group where there was no confounding by comorbidities or medication use. This was also a multi-ethnic cohort, reflecting the cultural and linguistic diversity of the wider Australian population [66], which is important in VDBP studies, since earlier studies were conducted mostly in Caucasian cohorts, despite knowledge of the interplay between ethnicity, genetics and the vitamin D metabolic system [67]. Vitamin D metabolites and VDBP were measured earlier in pregnancy than most previous studies, which enabled the assessment of longitudinal relationships between these biomarkers and pregnancy outcomes. We also adjusted for potential confounding by age, BMI, and ethnicity as well as parity or previous history of GDM, all of which have seldom been considered in previous studies. We used polyclonal assays which are considered more reliable in measuring VDBP, particularly in ethnically diverse cohorts [17]. This is because polyclonal assays are less affected by VDBP genotype polymorphisms compared with the more widely used monoclonal assays, since the latter technique relies on recognising an epitope near the polymorphic region of VDBP, which has different affinities for the various VDBP haplotypes [17].

## 5. Conclusions

In summary, the present study provides a valuable contribution to the sparse literature on VDBP in pregnancy. As the prevalence of GDM continues to rise in line with increasing maternal obesity, further assessments of lifestyle factors including nutritional status have become paramount and can help inform clinical and public health actions in this area. Our findings suggest a novel association between maternal circulating VDBP concentrations in early pregnancy and GDM, and support previous reports of an association between total 25(OH)D and GDM, as well as the length of gestation. These findings are in agreement with genetic studies linking VDBP with GDM and add to this evidence by showing a relationship with circulating VDBP concentrations. Assessing circulating VDBP may offer a simpler and more cost-effective means for risk prediction and prevention on a population scale. However, to determine the potential utility of these metabolites in predicting and preventing conditions such as GDM, future studies should incorporate: large-scale and adequately powered sample sizes; a diverse representative cohort with ethnic and genetic assessments; frequent sampling to track metabolite concentrations using direct gold-standard measures (polyclonal, LC/MS, and ELISA assays for VDBP, total and free 25(OH)D, respectively); and an assessment of lifestyle factors including sun exposure and vitamin D intake during pregnancy. Such studies can clarify the mechanistic pathways by which the vitamin D system interacts with the maternofetal axis and whether manipulation of these pathways can improve pregnancy outcomes.

## Figures and Tables

**Table 1:** Criteria for the inclusion and exclusion of participants in the HLP and CPO cohorts.

Inclusion Criteria		Exclusion Criteria	
HLP Cohort	CPO Cohort	HLP Cohort	CPO Cohort
Age ≥18 years	Age 18 ≤ 40 years	Age <18 years	Age <18 or >40 years
Singleton pregnancy		Multiple pregnancy	
High risk of GDM (scoring ≥3 on risk prediction tool [19])	Low-risk pregnancy (based on medical/obstetric history)	Known pre-existing diabetes diagnosis (any diabetes)	High-risk pregnancy (requiring care in high-risk clinic)
≤15 weeks gestation at recruitment	10–20 weeks gestation (±1 week) at recruitment	Other chronic medical conditions precluding participation	Models of care outside tertiary public hospital care (e.g., private, shared, or GP/midwife care)
English speaking		Non-English Speaking	
Overweight/obese: BMI ≥25 kg/m^2^ (or ≥23 kg/m^2^ in high-risk ethnicities) or ≥30 kg/m^2^	Attending Monash Health for labour	Morbidly obese (BMI ≥45 kg/m^2^)	Use of creatine supplements in pregnancy

Abbreviations: **HLP**, healthy lifestyle in pregnancy; **CPO**, creatine and pregnancy outcomes; **GDM**, gestational diabetes mellitus; **BMI**, body mass index; **GP**, general practice.

**Table 2:** Data collected at different gestational timepoints in the HLP and CPO pregnancy cohorts.

Timepoint (Gestation)	Data Collected in Both Cohorts	Methods
<20 weeks	Demographic characteristics: age, ethnicity, parity, smoking status, medical history	Demographic questionnaires
	BMI, weight, height	Weight (kg)/height (m)^2^ measured directly or calculated from SMR
	Fasting plasma glucose; serum/plasma total 25(OH)D, albumin, VDBP	Fasting venous blood samples and commercial assays for plasma glucose and total 25(OH)D; albumin and VDBP measured in bio-banked samples by Monash Pathology and Hudson laboratory (detailed below)
26–28 weeks	GDM diagnosis, fasting glucose, 1 h glucose post-OGTT, and 2 h glucose post-OGTT.	Data retrieved from routine OGTTs recorded on BOS or SMR. GDM diagnosed based on 2014 ADIPS criteria: FBG 5.1 or over and/or 2 h glucose 8.5 or over (mmol/L)
During pregnancy/at birth	Maternal outcomes and complications (including pre-eclampsia, pregnancy-induced hypertension, preterm birth)	Data retrieved from BOS and SMR

Abbreviations: **25(OH)D**, 25-hydroxyvitamin D; **ADIPS**, Australasian Diabetes in Pregnancy Society; **BMI**, body mass index; **BOS**, Birthing Outcomes System; **FBG**, fasting blood glucose; **GDM**, gestational diabetes; **HLP**, healthy lifestyle in pregnancy; **CPO**, creatine and pregnancy outcomes; **OGTT**, oral glucose tolerance test; **SMR**, scanned medical records; **VDBP**, vitamin D binding protein.

**Table 3:** The sample demographic, anthropometric, and biochemical characteristics and maternal pregnancy outcomes.

Variable	Mean ± SD or *n* (%)
Maternal age (years)	31.4 ± 4.2
***Parity***	
Primiparous	133 (46.0)
2	106 (36.7)
3	39 (13.5)
4	11 (3.8)
***Ethnicity***	
Caucasian	174 (57.4)
South East and North East Asian	37 (12.2)
Southern and Central Asian	69 (22.8)
Other ^a^	23 (7.6)
Past history of GDM	13 (4.3)
BMI (kg/m²)	26.8 ± 5.9
Gestational weight gain (kg, at 28 weeks)	7.4 ± 3.6
***Vitamin D metabolites***	
Total 25(OH)D, nmol/L (ng/mL)	54.8 ± 20.2 (22.0 ± 8.1)
Free 25(OH)D (pg/mL)	5.6 ± 4.7
Bioavailable 25(OH)D (nmol/L)	4.4 ± 3.1
VDBP (µg/mL)	364.7 ± 126.1
***Albumin (g/L)***	36.9 ± 4.1
***Glycaemic measures***	
FBG at baseline (< 20 weeks) (mmol/L)	4.6 ± 0.6
FBG OGTT (26–28 weeks) (mmol/L)	4.5 ± 0.5
1 h OGTT (26–28 weeks) (mmol/L)	7.6 ± 1.9
2 h OGTT (26–28 weeks) (mmol/L)	6.3 ± 1.5
***Pregnancy outcome***	
Gestational diabetes mellitus	55 (19.4)
Pre-eclampsia	10 (3.4)
Placental abnormality	102 (48.1)
Gestation at delivery (weeks)	39.1 ± 2.0
Preterm birth	16 (5.4)
Premature rupture of membranes	42 (20.4)
Caesarean section	75 (25.5)

Data reported as the mean ± standard deviation or frequency *n* (%); ^a^ Other represents African, Middle-Eastern, European, South American and Polynesian. Abbreviations: GDM, gestational diabetes mellitus; T2DM, type 2 diabetes mellitus; BMI, body mass index; 25(OH)D, 25 hydroxyvitamin D; VDBP, vitamin D binding protein; FBG, fasting blood glucose; OGTT, oral glucose tolerance test.

**Table 4:** Pearson correlations between vitamin D metabolites and maternal characteristics and glycaemic measures.

Variable	VDBP		Total 25(OH)D *		Free 25(OH)D *		Bioavailable 25(OH)D *	
	*r*	*p*	*r*	*p*	*r*	*p*	*r*	*p*
***Maternal characteristics***								
Age (years)	−0.24	**<0.01**	0.11	0.07	0.22	**<0.01**	0.24	**<0.01**
BMI (kg/m²)	−0.12	**0.047**	−0.26	**<0.001**	−0.11	0.08	−0.09	0.1
***Glycaemic measures***								
FBG (<20 weeks; mmol/L)	−0.02	0.7	−0.06	0.3	−0.003	0.9	−0.02	0.7
FBG OGTT (26–28 wks; mmol/L)	−0.11	0.08	−0.20	**<0.001**	−0.07	0.3	−0.05	0.4
1 h OGTT (26–28 weeks; mmol/L)	−0.17	**0.007**	−0.20	**0.001**	−0.02	0.8	−0.02	0.8
2 h OGTT (26–28 weeks; mmol/L)	−0.18	**0.003**	−0.16	**0.008**	0.008	0.9	0.001	0.9

All the data were assessed using Pearson correlations and are reported as the correlation coefficients (*r*) with the corresponding *p*-values (*p*). * Skewed variables were logarithmically transformed to the base 10 prior to analysis. Abbreviations: **BMI**, body mass index; **FBG**, fasting blood glucose; **OGTT**, oral glucose tolerance test; **VDBP**, vitamin D binding protein. Bold: denotes statistical significance at *p* < 0.05.

**Table 5:** Univariable associations between vitamin D metabolites and maternal outcomes.

Variable	VDBP		Total 25(OH)D		Free 25(OH)D		Bioavailable 25(OH)D	
	*β* or OR	*p*	*β* or OR (95%CI)	*p* *	β or OR	*p* *	*β* or OR (95%CI)	*p* *
	(95%CI)				(95%CI)			
GDM	0.98	**0.015**	0.98	**0.04**	0.99	0.9	1.01	0.9
	(0.97, 0.99)		(0.97, 0.99)		(0.93, 1.06)		(0.92, 1.10)	
PIH	1	0.5	1.03	0.06	1.02	0.3	1.04	0.3
	(0.99, 1.00)		(1.00, 1.06)		(0.91, 1.13)		(0.90, 1.22)	
Pre-eclampsia	0.99	0.1	1	0.8	1.06	0.09	1.12	0.08
	(0.99, 1.00)		(0.97, 1.03)		(0.98, 1.14)		(0.99, 1.24)	
Placental abnormalities	1	0.3	0.99	0.2	0.94	0.2	0.94	0.5
	(0.99, 1.00)		(0.98, 1.01)		(0.87, 1.02)		(0.85, 1.03)	
Gestation ^†^	0.001 ^†^	0.5	0.02 ^†^	**0.002**	0.02 ^†^	0.07	0.05 ^†^	0.07
	(−0.001, −0.003)		(0.004, 0.03)		(−0.03, 0.07)		(−0.02, 0.13)	
Preterm birth	0.99	0.3	0.98	0.1	0.88	0.4	0.89	0.6
	(0.99, 1.00)		(0.95, 1.01)		(0.70, 1.10)		(0.69, 1.15)	
PROM	1	0.8	0.99	0.5	0.94	0.4	0.94	0.6
	(0.99, 1.00)		(0.98, 1.01)		(0.84, 1.06)		(0.81, 1.08)	

Data were analysed using the general linear or simple logistic regression models for the continuous and binary outcomes, respectively, and the results are reported as beta coefficients **^†^** or odds ratios with 95% confidence intervals and the corresponding *p*-values; * *p*-values represent the significance of the analyses after vitamin D metabolite data were logarithmically transformed to the base 10 to approximate normality. Abbreviations: **25(OH)D**, 25-hydroxyvitamin D; **OR**, odds ratio; **VDBP**, vitamin D binding protein; **GDM**, gestational diabetes mellitus; **PIH**, pregnancy-induced hypertension; **PROM**, premature rupture of membranes. Bold: denotes statistical significance at *p* < 0.05.

**Table 6:** Multivariable regression analyses of the relationships between vitamin D metabolites and the pregnancy outcomes after adjustment for maternal covariates.

Dependent Variable	Model	VDBP				Total 25(OH)D				Free 25(OH)D				Bioavailable 25(OH)D			
		*β*	SE	*R* ^2^	*p*	*β*	SE	*R* ^2^	*p*	*β*	SE	*R* ^2^	*p*	*β*	SE	*R* ^2^	*p*
GDM	+ age	−0.003	0.001	0.05	**0.04**	−0.02	0.01	0.05	**0.01**	−0.02	0.04	0.03	0.4	−0.03	0.05	0.03	0.5
	+ BMI	−0.003	0.001	0.06	0.05	−0.01	0.01	0.05	**0.04**	−0.01	0.04	0.04	0.7	−0.01	0.05	0.04	0.7
	+ ethnicity	−0.003	0.001	0.07	**0.03**	−0.01	−0.03	0.01	0.1	0.002	0.04	0.05	0.9	0.01	0.05	0.05	0.9
Pregnancy-induced hypertension	+ age	0.001	0.002	0.004	0.5	0.03	0.01	0.05	0.06	0.02	0.06	0.001	0.3	0.05	0.08	0.003	0.3
	+ BMI	0.001	0.003	0.002	0.7	0.02	0.02	0.01	0.2	0.001	0.07	0.0003	0.5	0.02	0.10	0.001	0.5
	+ ethnicity	0.001	0.003	0.05	0.7	0.01	0.02	0.05	0.5	−0.02	0.09	0.05	0.8	−0.01	0.11	0.05	0.9
Pre-eclampsia	+ age	−0.003	0.003	0.06	0.3	0.001	0.02	0.05	0.6	0.05	0.07	0.10	0.2	0.10	0.06	0.07	0.2
	+ BMI	−0.003	0.003	0.09	0.4	0.007	0.02	0.08	0.5	0.07	0.04	0.11	0.2	0.10	0.06	0.11	0.1
	+ ethnicity	−0.003	0.003	0.09	0.4	0.007	0.02	0.08	0.9	0.07	0.04	0.11	0.1	0.10	0.06	0.11	0.1
Length of gestation	+ age	0.0004	0.001	0.003	0.7	0.02	0.01	0.03	**0.001**	0.03	0.03	0.007	**0.039**	0.07	0.04	0.01	**0.03**
	+ BMI	0.0004	0.001	0.003	0.7	0.02	0.01	0.03	**0.001**	0.03	0.03	0.007	**0.03**	0.07	0.04	0.01	**0.03**
	+ ethnicity	0.0004	0.001	0.03	0.6	0.02	0.01	0.05	**0.006**	0.02	0.03	0.03	0.1	0.05	0.04	0.03	0.1
Preterm birth	+ age	−0.002	0.002	0.01	0.3	−0.02	0.01	0.02	0.1	−0.14	0.12	0.02	0.4	−0.13	0.13	0.01	0.6
	+ BMI	−0.002	0.002	0.01	0.3	−0.02	0.02	0.02	0.09	−0.14	0.12	0.02	0.4	−0.13	0.14	0.01	0.5
	+ ethnicity	−0.002	0.002	0.01	0.3	−0.02	0.01	0.02	0.1	−0.14	0.13	0.02	0.5	−0.12	0.14	0.01	0.6
PROM	+ age	−0.0003	0.002	0.0003	0.8	−0.01	0.01	0.01	0.4	−0.06	0.06	0.01	0.5	−0.06	0.07	0.01	0.6
	+ BMI	−0.0004	0.002	0.001	0.8	−0.01	0.01	0.01	0.3	−0.07	0.06	0.01	0.4	−0.06	0.08	0.01	0.5
	+ ethnicity	−0.0004	0.002	0.001	0.8	−0.01	0.01	0.01	0.3	−0.07	0.07	0.01	0.4	−0.07	0.08	0.02	0.6

Data are presented as unstandardized beta coefficients (*β*) with the corresponding standard error (SE), and *R*^2^ (or pseudo *R*^2^ for the logistic regression of binary outcomes). The plus signs indicate the addition of each variable to the model (e.g., for GDM, the first row is a model adjusted for age only, the second row is adjusted for age and BMI, and so on). Abbreviations: **GDM**, gestational diabetes mellitus; **PROM**, premature rupture of membranes. Bold: denotes statistical significance at *p* < 0.05.
